# Supplementary material for: Effects of Voluntary Wheel Running Exercise on Chemotherapy-Impaired Cognitive and Motor Performance in Mice
Source: Int J Environ Res Public Health. 2023 Apr 3;20(7):5371. doi: 10.3390/ijerph20075371 (PMC10094707; doi:10.3390/ijerph20075371)
Supplement: Supplementary file 1 [file ijerph-20-05371-s001.zip › ijerph-2255640-supplementary.pdf]

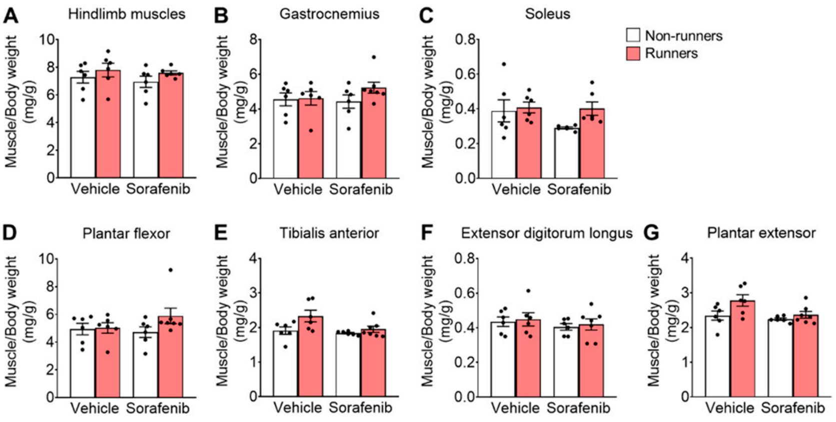

Supplementary Figure S1: Neither voluntary running nor sorafenib administration affected skeletal muscle weight. *Neither sorafenib administration nor voluntary running exercise affected muscle weight per body weight in the* ***(A)*** *hindlimbs,* ***(B)*** *gastrocnemius,* ***(C)*** *soleus,* ***(D)*** *plantar flexor,* ***(E)*** *tibialis anterior,* ***(F)*** *extensor digitorum longus, and* ***(G)*** *plantar extensor. Results were expressed as mean ± SEM. n = 6 per group.*

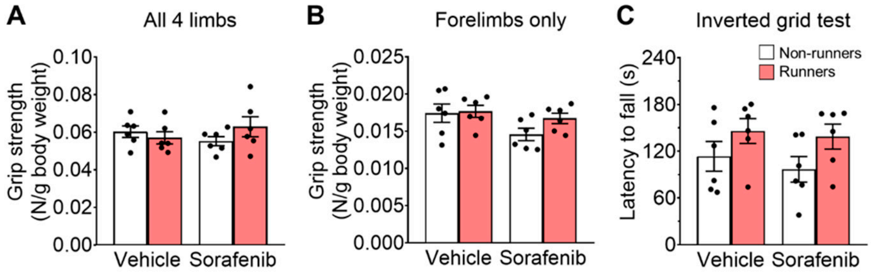

Supplementary Figure S2: Effect of sorafenib administration and voluntary wheel running exercise on skeletal muscle strength. *Neither sorafenib administration nor voluntary running exercise affected the grip strength of* ***(A)*** *all four limb muscles and* ***(B)*** *forelimbs only in the grip strength test.* ***(C)*** *Also, neither treatment affected the performance in the inverted grid test. Results were expressed as mean ± SEM. n = 6 per group.*
